# Suppression Mechanism of Early-Age Autogenous Shrinkage Cracking in Low Water-to-Binder Ratio Cement-Based Materials Incorporating Ground Granulated Blast-Furnace Slag and Silica Fume

**DOI:** 10.3390/ma19010131

**Published:** 2025-12-30

**Authors:** Shuangxi Li, Guanglang You, Gang Yu, Chunmeng Jiang, Xinguang Xia, Dongzheng Yu

**Affiliations:** 1 College of Hydraulic and Civil Engineering, Xinjiang Agricultural University, Urumqi 830052, China; 2 Xinjiang Tianze Engineering Management Co., Ltd., Shihezi 832003, China; 3 Xinjiang Yuance Engineering Technology Co., Ltd., Changji 831100, China

**Keywords:** blended GGBS-SF, low-shrinkage concrete, crack-resistant concrete, high-strength concrete

## Abstract

In hydraulic structures such as water control projects, spillway tunnels, and overflow dams that are subjected to high-velocity flow erosion, Concrete is required to exhibit high resistance to abrasion and cracking. While low water-to-binder ratio concrete can meet strength requirements, its inherent high shrinkage propensity often leads to cracking, seriously compromising long-term safety and durability under severe operating conditions. To address this engineering challenge, this study focuses on optimizing concrete performance through the synergistic combination of slag (GGBS) and silica fume (SF). This study systematically investigated the effects of incorporating GGBS (20–24%) and SF (6–10%) in a low water-to-binder ratio system with a fixed 70% cement content on key concrete properties. The evaluation was conducted through comprehensive tests including compressive strength, drying shrinkage, autogenous shrinkage, and hydration heat analysis. The results demonstrate that the blended system successfully achieves a synergistic improvement in both “high strength” and “low cracking risk.” Specifically, the incorporation of silica fume significantly enhances the compressive strength at all ages, providing a solid mechanical foundation for resisting high-velocity flow erosion. More importantly, compared to the pure cement system, the blended system not only delays the onset but also reduces the rate of early-age shrinkage, and lowers its ultimate autogenous shrinkage value. This characteristic is crucial for controlling the combined effects of thermal and shrinkage stresses from the source and preventing early-age cracking. Simultaneously, hydration heat analysis reveals that the blended system retards the heat release process, which helps mitigate the risk of thermal cracking. This study elucidates the regulatory mechanism of the GGBS-SF combination and provides a critical mix design basis and theoretical support for producing high-strength, high-abrasion-resistant, and low-shrinkage concrete in high-velocity flow environments, offering direct practical implications for engineering applications.

## 1. Introduction

The development of high-performance and ultra-high-performance concrete (UHPC) represents a significant advancement in modern construction materials, offering exceptional mechanical properties and durability [1]. A fundamental characteristic of these concrete types, particularly ultra-high-performance concrete (UHPC), is a low water-to-binder (*w*/*b*) ratio, often below 0.25. In this study, a *w*/*b* of 0.20 was used, which, while at the lower end of the spectrum for such materials, is essential for achieving high compactness and strength [2,3]. However, this very attribute introduces a set of critical challenges, primarily related to the internal moisture environment and the kinetics of cement hydration. The reduced water content, combined with the consumption of water during the hydration process and the formation of a dense, low-permeability microstructure, leads to significant autogenous shrinkage [4]. This early-age volume change, occurring under isothermal and sealed conditions, can induce high internal stresses and lead to microcracking, thereby compromising the long-term durability, structural integrity, and service life of concrete structures [5].

Concurrently, the hydration of Portland cement is an exothermic process. In massive concrete elements, such as foundations, bridge piers, and thick slabs, the heat generated by cement hydration is difficult to dissipate, leading to a significant temperature rise at the core [6]. The subsequent non-uniform cooling and thermal contraction, often restrained by external or internal boundaries, can result in thermal cracking, which provides pathways for the ingress of aggressive agents [7]. Mitigating both autogenous shrinkage and heat of hydration is therefore paramount for the successful application of low *w*/*b* ratio concrete, especially in large-scale or critical infrastructure projects.

A widely adopted strategy to address these issues is the partial replacement of ordinary Portland cement (OPC) with supplementary cementitious materials (SCMs) [8]. Among various SCMs, ground granulated blast-furnace slag (GGBS) and silica fume (SF) are two of the most effective and commonly used materials [9]. GGBS, a by-product of the iron industry, reacts pozzolanically and, more importantly, hydraulically in the presence of cement hydration products like calcium hydroxide (CH) [10]. Its incorporation is known to reduce the peak hydration temperature and decelerate the rate of heat evolution, primarily due to its slower reaction kinetics compared to OPC [11,12]. Furthermore, the dilution effect and the subsequent slower strength development can influence the shrinkage behavior [13].

Silica fume, an ultrafine by-product from silicon and ferrosilicon alloy production, is highly pozzolanic [14,15]. Its primary role is to fill the interstitial spaces between cement grains and to react with CH to form additional calcium-silicate-hydrate (C-S-H) gel, dramatically refining the pore structure and enhancing mechanical strength and durability [16,17]. However, this very process of pore refinement and the high consumption of water for the pozzolanic reaction can exacerbate the self-desiccation process, potentially increasing the driving force for autogenous shrinkage in low *w*/*b* ratio systems [18,19].

The concept of “binary blending” (using one SCM) has been extensively studied, but the “ternary blending” of OPC, GGBS, and SF has garnered increasing interest for its potential synergistic effects [20,21]. It is hypothesized that the combination of these two SCMs can offer a more balanced approach: the GGBS mitigates the heat of hydration and may contribute to a more favorable long-term pore structure, while the SF provides early-age strength and ultra-high performance [22,23]. The interaction between the slower-reacting GGBS and the highly reactive SF could potentially modify the hydration process, the evolution of the microstructure, and the development of internal relative humidity, thereby influencing the autogenous deformation [24]. Nevertheless, the exact nature of this synergy and its quantitative impact on the combined challenges of heat generation and autogenous shrinkage in very low *w*/*b* ratio systems remains a subject of active research, with some studies reporting mitigating effects and others indicating complex, mix-dependent interactions [25,26].

Therefore, a comprehensive understanding of the coupled effects of GGBS and silica fume on the hydration thermodynamics, reaction kinetics, and autogenous shrinkage development is crucial for the optimized design of sustainable and crack-resistant high-performance concrete. This study aims to systematically investigate the heat of hydration and autogenous shrinkage of very low *w*/*b* ratio concrete incorporating binary and ternary blends of GGBS and silica fume. By elucidating the underlying mechanisms, this research seeks to provide guidance for tailoring concrete mixtures to mitigate early-age volume changes and thermal cracking, enhancing the durability and longevity of modern concrete infrastructure.

Therefore, this study is designed to systematically elucidate the phase-specific synergistic mechanisms of GGBS and SF on hydration and shrinkage in low *w*/*b* systems. By correlating macroscopic thermodynamic and deformation data with the underlying microstructural roles of each SCM, it seeks to fill these identified gaps and provide a solid theoretical basis for designing high-performance concrete with low shrinkage and reduced thermal cracking risk.

## 2. Experimental Materials

### 2.1. Materials

1. Cement

The cement used in this experiment was Type P.O 42.5R Ordinary Portland Cement (OPC), manufactured and produced by Xinjiang Tianshan Materials Co., Ltd. (Urumqi, China). The production date was 16 March 2025. Its chemical composition is presented in Table 1, and the physical and mechanical properties are shown in Table 2.

2. GGBS

GGBS used in this test was an S75-grade slag powder produced by Baoxin Shengyuan Building Materials Co., Ltd. (Urumqi, China). The chemical composition of the slag is presented in Table 3.

3. SF

The silica fume was sourced from Lanzhou, Gansu Province, and its composition is provided in Table 4.

4. Superplasticizer

A polycarboxylate ether-based high-range water-reducing admixture (PCE superplasticizer), specifically designed for ultra-high-performance concrete (UHPC), was employed. The product (Commercial Model: JFTR-PCE (UHPC-grade), Sichuan Dongrun Baisheng New Materials Co., Ltd., Chengdu, China) is characterized by its high dispersion efficiency in low water-binder ratio systems. While the precise polymer architecture (e.g., molecular weight, exact side-chain length) is proprietary to the manufacturer, the supplier specifies that it is a comb-type copolymer with polyoxyethylene (PEO) side chains optimized for UHPC applications. Key supplied properties include a solid content of 25.2%, a density of 1.025 g/cm^3^, and very low contents of chloride (0.03%).

The physical properties of the superplasticizer are shown in Table 5.

5. Water

The experiments utilized standard laboratory tap water.

6. Coarse Aggregate

Naturally available crushed stone, locally sourced from Xinjiang, was employed as the coarse aggregate.

7. Fine Aggregate

Natural river sand from Xinjiang, with a fineness modulus of 2.86 and well-graded particle size distribution, was utilized as the fine aggregate.

The chemical compositions of the cement (Table 1 and Table 2), GGBS (Table 3), and silica fume (Table 4) used in this study were obtained from the certificates of analysis provided by the material manufacturers.

8. Mixture Proportioning Design

The concrete mix design is shown in Table 6. This study employed a ternary binder system consisting of cement, ground granulated blast-furnace slag (GGBS), and silica fume (SF) for mixture proportioning design to investigate the effects of different cementitious compositions on the compressive strength, heat of hydration, autogenous shrinkage, and drying shrinkage of cement-based materials with an ultra-low water-to-binder ratio. The designed water-to-binder ratio (*w*/*b*) was maintained at 0.20, with a binder-to-sand ratio of 0.45. A superplasticizer dosage of 2.0% by mass of binder and a total binder content of 860 kg/m^3^ were utilized. Subsequently, specimen casting, curing, and corresponding experimental testing were conducted in strict accordance with the predetermined experimental protocol. All required experiments were conducted in accordance with the relevant standards, using a minimum of three test specimens per condition. Outliers were excluded to prevent random anomalies in the data.

### 2.2. Experimental Methods and Testing Apparatus

1. Specimen Preparation

The constituent materials were precisely weighed according to the designated mix proportions and subsequently introduced into a forced-action concrete mixer. An initial dry mixing period of 180 s was conducted to achieve homogeneous blending of the cementitious materials, fine aggregate, and coarse aggregate. The pre-weighed superplasticizer was first dissolved in the pre-measured tap water under constant agitation. The resulting solution was then added to the mixer in two stages: approximately two-thirds of the solution was initially incorporated and mixed until the mixture achieved a uniform sandy appearance. Subsequently, the remaining one-third of the solution was added, followed by an additional wet mixing duration of 240 s until a homogeneous, flowable consistency was obtained. The freshly mixed concrete was immediately transferred to appropriate molds corresponding to the specific testing requirements.

2. Compressive Strength Testing

Compressive strength represents the most fundamental and critical mechanical property of materials, serving as a key indicator for evaluating the degree of specimen deterioration. Uniaxial compressive strength tests were conducted using a CSS-44100 electronic universal testing machine (Xinjiang Yuance Engineering Technology Co., Ltd., Changji 831100, China). The freshly mixed concrete from each group was cast into 100 mm × 100 mm × 100 mm molds. A vibrating table was employed to eliminate entrapped air bubbles from the molds. Subsequently, the specimens were covered with plastic membrane and cured in a standard curing chamber (20 °C, 98% RH) for 24 h before demolding. After confirming the absence of defective specimens (determined by the absence of corner breakage during demolding), all specimens were returned to the standard curing chamber for continued curing. The compressive strength tests were performed at curing ages of 3, 7, and 28 days under controlled laboratory conditions of 20 ± 2 °C.

3. Autogenous Shrinkage Test

The freshly mixed concrete from each formulation was cast into 100 mm × 100 mm × 515 mm molds. Prior to concrete placement, to minimize moisture exchange, the specimens, while still in their molds, were sealed with two layers of low-density polyethylene (LDPE) film (0.05 mm). Machine oil was applied as a lubricant at two specific interfaces: (1) between the inner surface of the mold and the first layer of film, and (2) between the first and the second layers of film. This procedure ensured a complete, void-free seal by eliminating air gaps and allowing the film to conform tightly without restraining the specimen. Target plates were then fixed at both ends of the mold, ensuring a gauge length exceeding 400 mm. After concrete placement, the molds were immediately sealed following consolidation on a vibrating table to eliminate entrapped air. Displacement sensors were zeroed at both ends immediately after sealing to initiate testing before initial setting. Measurements were conducted using an NELD-ES730 (6-channel) non-contact concrete shrinkage deformation instrument (Xinjiang Yuance Engineering Technology Co., Ltd., Changji 831100, China) under constant temperature (20 ± 2) °C and humidity (50 ± 5) % conditions. The test duration was 168 h with data recorded at 1-h intervals.

4. Drying Shrinkage Test

The drying shrinkage test was conducted in accordance with the Chinese industry standard GB/T 29417-2012 “Cement Mortar Dry Shrinkage Test Method” [27]. The experimental procedure was as follows: Steel molds measuring 25 mm × 25 mm × 280 mm were used for specimen preparation. Prior to molding, the molds were thoroughly cleaned and all surrounding templates were tightly assembled with the base. A uniform layer of machine oil was applied to the inner walls of the mold. The gauge pins were carefully cleaned and embedded into the designated holes in the mold, rotating them left and right to ensure proper seating and alignment. The prepared mortar was then placed into the mold in two layers, with each layer compacted and leveled using a small trowel and square tamping rod. The filled molds were transferred to the curing room and demolded after 24 h of curing. After demolding, the specimens were numbered and placed in a drying shrinkage maintenance chamber. The drying shrinkage rate of the specimens was determined using a length comparator, with measurements recorded at 24-h intervals.

5. Heat of Hydration Test

Isothermal calorimetry was employed to determine the heat of hydration using a TAM Air isothermal calorimeter. To minimize experimental error, all materials were preconditioned at 20 °C prior to testing. Freshly mixed paste was immediately transferred to the instrument’s channels and maintained at 20 °C for 168 h of continuous monitoring.

6. X-ray Diffraction (XRD) Analysis

To compare the phase composition changes in concrete with varying GGBS/SF replacement ratios, surface samples were extracted from specimens at different curing ages. Powder samples were obtained by grinding from the edge toward the center of cubic specimens (avoiding coarse aggregates), followed by drying and sieving through an 80 μm square-mesh sieve. XRD analysis was performed using a Bruker D8 Advance diffractometer (Shanghai, China) operated at 40 kV and 40 mA, with a scanning rate of 10°/min over a 2θ range of 10–70°.

7. SEM microstructure

Concrete specimens (10 mm × 10 mm × 20 mm) were prepared for microstructural observation of hydration products using scanning electron microscopy. quantify chemical compositions, and determine calcium-to-silicon ratios.

**Figure 1:**
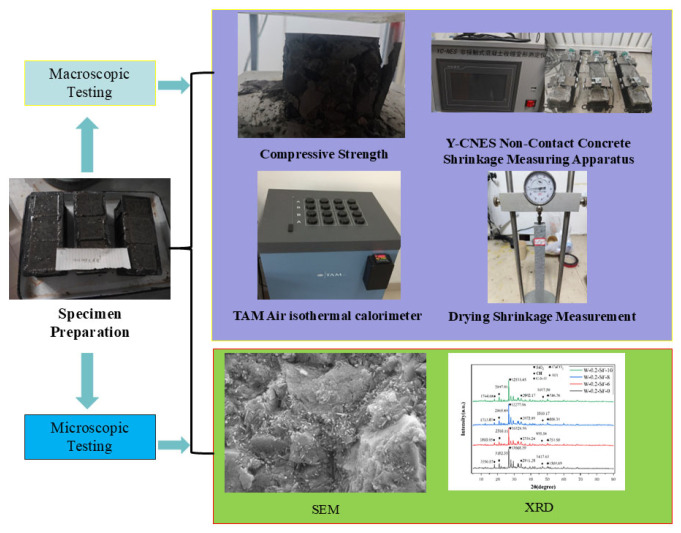
Experimental Procedure.

The test procedure is illustrated in Figure 1.

## 3. Results and Discussion

### 3.1. Analysis of Mechanical Properties of GGBS-SF Composite Concrete

**Figure 2:**
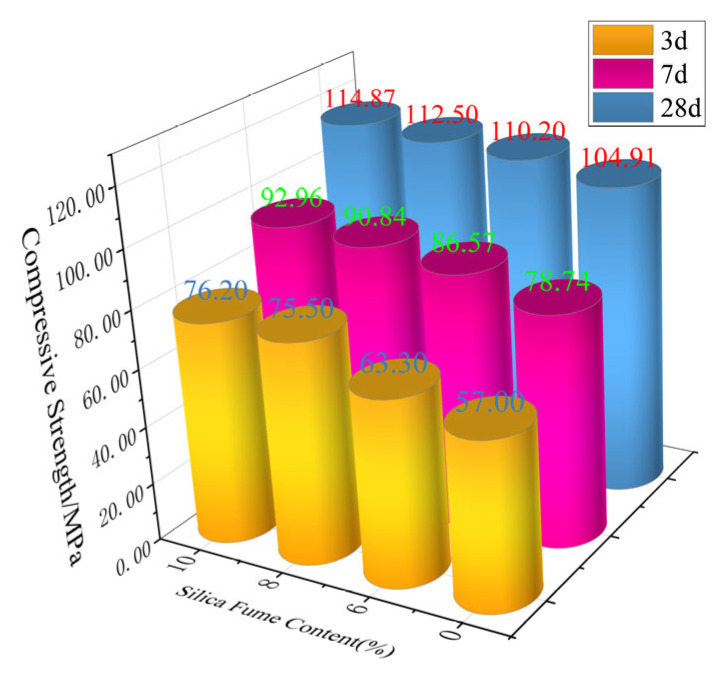
Compressive Strength at Various Ages as a Function of Silica Fume Content.

The compressive strength test results for four groups of specimens at different ages were determined based on the average of three measurements. If any individual value deviated by more than 15% from the median value, the median was adopted as the test result. The results clearly demonstrate the influence of varying GGBS and SF contents on concrete compressive strength, revealing that: The compressive strength progressively increased with higher SF replacement ratios; At 6% SF replacement, the early-age strength development rate was significantly faster than the other two groups, though this difference diminished at later ages; The growth rates between 8% and 10% SF replacement groups showed no significant difference. The compressive strength of concrete at different curing ages is as shown in Figure 2.

Regarding 3-day compressive strength, SF played a crucial role in early-strength development through its reaction with free Ca(OH)_2_, which promoted C_2_S hydration and enhanced early-age strength. As SF content increased from 6% to 10%, the 3-day compressive strength showed an increasing trend. In contrast, GGBS requires an alkaline environment (provided by cement hydration products like Ca(OH)_2_) for pozzolanic reactions to develop strength. The limited availability of Ca(OH)_2_ at early ages resulted in reduced early strength, though this negative effect diminished when GGBS content decreased from 24% to 20%.

At 7 days, SF continued to contribute to strength development by densifying the microstructure, with higher replacement ratios generally providing greater strength enhancement. While GGBS pozzolanic activity increased somewhat at this stage, it remained less significant than at later ages. Lower GGBS contents corresponded to relatively higher 7-day strengths.

By 28 days, SF further enhanced compressive strength through pore-filling effects and microstructural densification, typically demonstrating greater improvement with higher replacement ratios within certain limits. Meanwhile, GGBS exhibited substantial pozzolanic reactivity, reacting with water and Ca(OH)_2_ to generate additional C-S-H gel that increased density and strength. Within the 20–24% replacement range, GGBS positively contributed to 28-day strength, with potentially higher strength at lower replacement ratios—though higher SF contents could compensate for strength reduction resulting from decreased GGBS content. In summary, the effects of SF and GGBS on compressive strength at different ages represent a complex interaction between their replacement ratios, mutual interactions, and other factors including w/b ratio and curing conditions. Generally, when incorporating varying proportions of SF and GGBS: SF primarily enhances both early and later age strength, GGBS plays a more significant role in later strength development, Decreasing GGBS content while increasing SF content proves beneficial for improving 3 d, 7 d, and 28 d compressive strength.

### 3.2. Analysis of Shrinkage Properties of GGBS-SF Composite Concrete

#### 3.2.1. Drying Shrinkage Analysis

Drying shrinkage refers to the contraction phenomenon resulting from moisture evaporation induced by humidity gradient differences between the internal and external environments when cement-based materials are exposed to low humidity conditions. In dry environments, mitigating shrinkage in high-strength concrete requires simultaneous consideration of both autogenous and drying shrinkage mechanisms.

The development rate of drying shrinkage was most rapid within the initial 0–7 days, primarily due to the abundant free water content in capillary pores and rapid moisture loss during this period. Between 7–12 days, the shrinkage rate decelerated but remained relatively significant. During this phase, SF contributed to shrinkage reduction through pore refinement and interfacial transition zone enhancement, while GGBS mitigated shrinkage via secondary hydration reactions that densified the pore structure. Beyond 12 days, the shrinkage progression continued at a markedly reduced rate owing to diminished free water content and minimized humidity differentials between the concrete interior and external environment.

**Figure 3:**
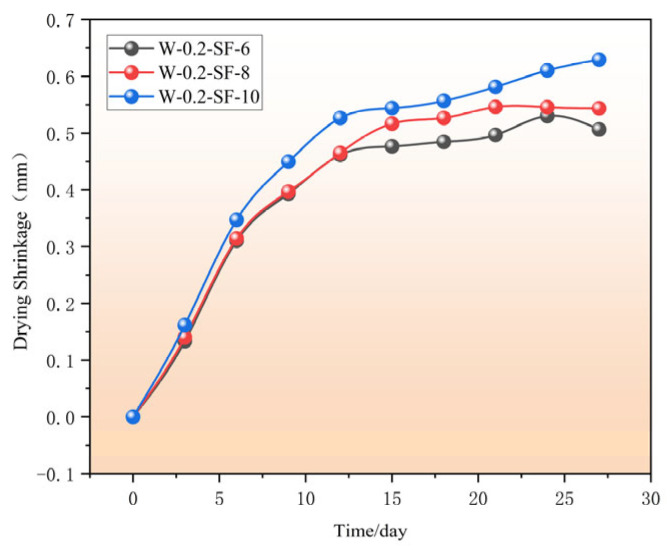
Drying Shrinkage Strain as a Function of Time.

As clearly demonstrated in Figure 3, the SF content significantly influenced the magnitude of drying shrinkage: specimens with 6%, 8%, and 10% SF replacement exhibited progressively increasing shrinkage values, with the minimum and maximum shrinkage observed at 6% and 10% SF replacement, respectively. After 12 days, the development of drying shrinkage values essentially enters a stable phase. This is because the primary hydration reactions of cement and silica fume are largely complete by this time, and the subsequent hydration process contributes only minimally to further drying shrinkage.

#### 3.2.2. Autogenous Shrinkage Analysis

Autogenous shrinkage refers to the volumetric contraction occurring during the hydration reaction stage after initial setting, primarily caused by the formation of calcium silicate hydrate (C-S-H) and calcium aluminate hydrate gels. This phenomenon may induce internal stress in concrete, particularly when internal moisture is insufficient.

**Figure 4:**
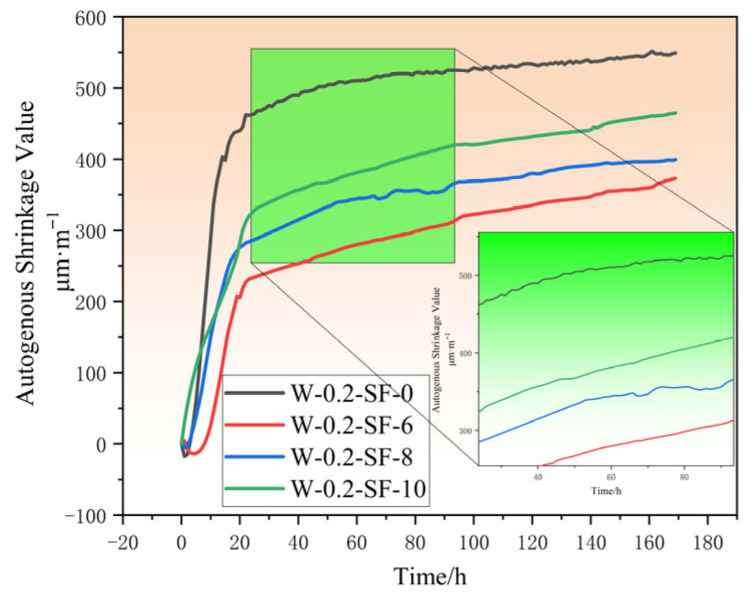
Autogenous Shrinkage Strain as a Function of Time for Different Concrete Mixtures.

As shown in Figure 4, the autogenous shrinkage development of the four concrete mixtures (W-0.2-SF-0, W-0.2-SF-6, W-0.2-SF-8, W-0.2-SF-10) consistently exhibited three distinct phases: expansion, rapid shrinkage, and slow shrinkage.

During the initial phase, concrete displayed volumetric expansion due to plastic settlement causing vertical contraction and lateral expansion, coupled with varying hydration behaviors of different binder combinations. The expansion duration for the four groups was 0–2 h, 0–5 h, 0–2 h, and 0 h, with peak expansion values of (negative indicates contraction) −17.33 μm/m, −13.62 μm/m, −5.21 μm/m, and 0 μm/m, respectively. In this phase, SF’s accelerated hydration and self-desiccation effects predominated, while GGBS only partially mitigated the shrinkage rate. Increasing SF content progressively reduced expansion magnitude, with the 10% SF mixture showing zero expansion.

The rapid shrinkage phase followed, with cumulative autogenous shrinkage at 24 h of 461.71 μm/m, 233.02 μm/m, 285.44 μm/m, and 324.01 μm/m for the respective groups, showing increasing shrinkage with higher SF content. Compared to the control group, shrinkage reductions were 49.5%, 38.18%, and 29.82% for the 6%, 8%, and 10% SF groups, respectively. This behavior resulted from SF’s dominant effects: higher SF content accelerated early hydration heat release, rapid moisture consumption, and earlier development of capillary pore negative pressure. The significant shrinkage periods were 2–24 h, 5–24 h, 2–24 h, and 0–24 h, with the 10% SF group exhibiting the earliest initiation.

After 24 h, the slow shrinkage phase commenced, divisible into 24–72 h and 72–168 h sub-phases. During 24–72 h, SF continued to drive shrinkage while GGBS’s restraining effect remained limited: SF-induced micro-pores maintained high capillary negative pressure, while GGBS hydration products only slightly reduced macropore negative pressure. In the 72–168 h period, GGBS’s hydration slowed but its structural effects became apparent: near-saturation of GGBS hydration reduced shrinkage rate, and pore-filling action created more uniform pore distribution. The 24% GGBS group showed lower shrinkage rate than the 20% group, though overall shrinkage remained SF-content dependent.

By 168 h, autogenous shrinkage values essentially stabilized at 549.29 μm/m, 372.99 μm/m, 399.54 μm/m, and 464.82 μm/m, representing reductions of 32.1%, 27.3%, and 15.4% compared to the control. The combination of 6% SF and 24% GGBS demonstrated the most significant effectiveness in reducing autogenous shrinkage.

### 3.3. Analysis of Hydration Heat in GGBS-SF Composite Concrete

**Figure 5:**
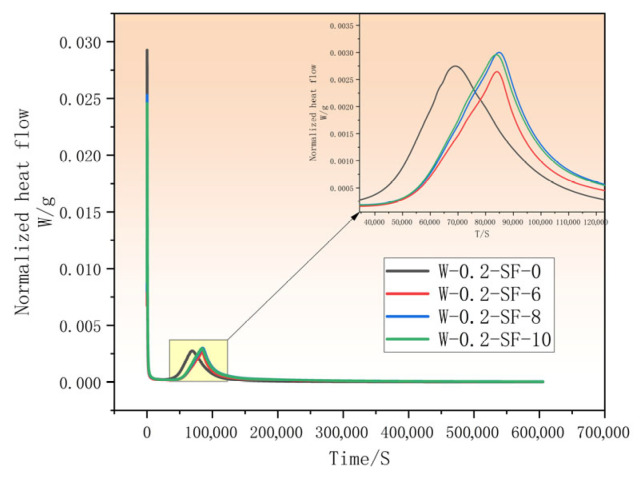
Rate of Heat Evolution as a Function of Time.

**Figure 6:**
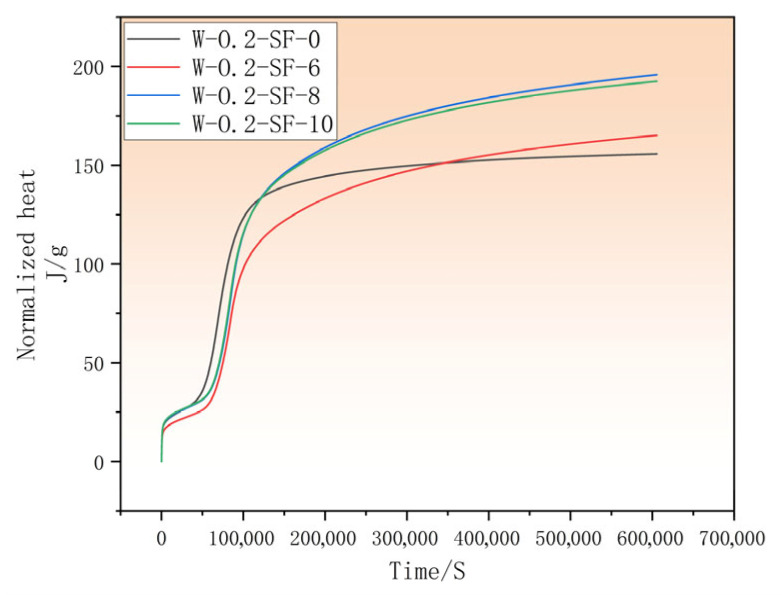
Cumulative Heat of Hydration at 7 Days.

The incorporation of varying proportions of GGBS and SF significantly influenced the heat evolution characteristics of concrete. As shown by the hydration rate in Figure 5 and the cumulative heat release in Figure 6, during the early reaction stage, all three blended mixtures exhibited lower heat release compared to the control group, with their main hydration peaks occurring later. The mixture with the lowest SF content maintained its lower heat release relative to the control for a substantially longer duration than the other two blends. However, ultimately all three blended mixtures demonstrated higher cumulative heat than the control.

Comprehensively, both SF and GGBS reduced early-age heat release. Comparative analysis indicates that higher GGBS content combined with lower SF dosage effectively suppressed early hydration heat. After the initial period, mixtures with higher SF content reacted earlier, resulting in an accelerated main hydration peak and shorter time to reach the control’s heat level. During the final reaction stage, GGBS contributed to heat reduction, making the 6% SF mixture exhibit the lowest heat among blends, though still higher than the control due to SF’s continuing effect. Furthermore, when SF content reached 8% and 10%, the heat evolution patterns became nearly identical, suggesting a limiting threshold for SF’s influence on hydration heat.

During the pre-induction period, all four mixtures (W-0.2-SF-0, W-0.2-SF-6, W-0.2-SF-8, W-0.2-SF-10) initiated rapid reaction immediately (0 s), reaching peak rates at 140 s, 180 s, 180 s, and 180 s, respectively. As shown in Figure 5, the corresponding peak heat evolution rates were 0.0293 W/g, 0.0237 W/g, 0.0253 W/g, and 0.0246 W/g, with pre-induction durations of 0.69 h, 0.62 h, 0.77 h, and 0.81 h. This brief but intense reaction phase resulted from rapid pore-filling by GGBS-SF and pozzolanic consumption of Ca(OH)_2_, the reaction kinetics may be delayed, possibly related to delayed Ca^2+^ release due to dilution and pozzolanic consumption.

Following the pre-induction period, an extended induction period commenced with negligible heat release. The induction periods began at 0.69 h, 0.62 h, 0.77 h, and 0.81 h, ending at 4.23 h, 6.76 h, 7.09 h, and 7.13 h, respectively, with durations of 3.54 h, 6.14 h, 6.32 h, and 6.32 h. The hydration progression during this period, primarily involving C_3_S and the tricalcium aluminate-gypsum system, significantly influenced subsequent performance development. Generally, GGBS-SF incorporation substantially extended the induction period, with W-0.2-SF-8 and W-0.2-SF-10 showing identical durations. This extension resulted from GGBS’s lower reactivity reducing cement-water contact area, while SF’s high surface area and reactivity consumed Ca(OH)_2_, creating competing effects that maintained the induction period at 6.32 h for the higher SF blends. Additionally, the heat evolution rates during induction were significantly lower than the control.

Upon concluding the induction period, hydration reaccelerated entering the acceleration period with a second heat peak. Although significantly increased, the second peak rates (0.002745 W/g, 0.002645 W/g, 0.003000 W/g, and 0.002958 W/g, respectively) remained lower than the initial peaks. The W-0.2-SF-6 mixture showed the lowest peak rate while W-0.2-SF-8 exhibited the highest, with both 8% and 10% SF mixtures exceeding the control’s peak rate. This behavior suggests that during this period, increased Ca(OH)_2_ from cement hydration activated GGBS’s secondary reactions, generating additional hydration products (ettringite and C-S-H) that sustained heat release and enhanced later-age strength. Simultaneously, SF’s high reactivity accelerated cement hydration through Ca(OH)_2_ consumption, enabling faster strength development.

The stabilization period following the acceleration phase marked the completion of major hydration reactions. Notably, the cumulative heat curves revealed that the control mixture ultimately exhibited lower total heat release than all blended mixtures. This indicates that GGBS-SF incorporation promoted continued secondary reactions, contributing to additional binder formation and thus enhancing long-term strength development.

### 3.4. Analysis of Microstructural Test Results for GGBS-SF Composite Concrete

**Figure 7:**
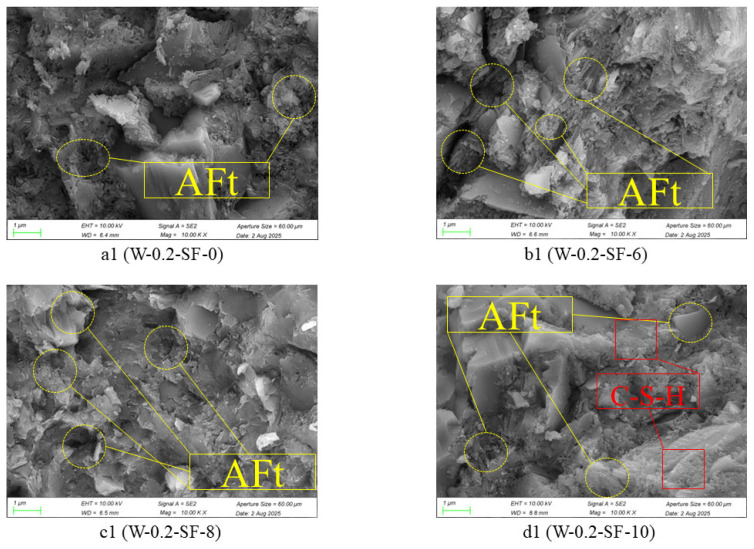
Microstructure at 3 Days.

Comparative analysis of the 3-day SEM images in Figure 7 reveals significant differences in the microstructural characteristics of pastes with different combination ratios. The high-SF mixture (W-0.2-SF-10) exhibited abundant dense C-S-H gel that tightly encapsulated cement and SF particles, resulting in well-connected microstructural continuity. In contrast, the high-GGBS mixture (W-0.2-SF-6) showed sparsely distributed C-S-H gel with numerous unhydrated GGBS particles, presenting a more porous microstructure compared to the high-SF mixture. All experimental mixtures demonstrated denser microstructures than the control (W-0.2-SF-0), with density increasing proportionally to SF content following the sequence: W-0.2-SF-0 < W-0.2-SF-6 < W-0.2-SF-8 < W-0.2-SF-10.

**Figure 8:**
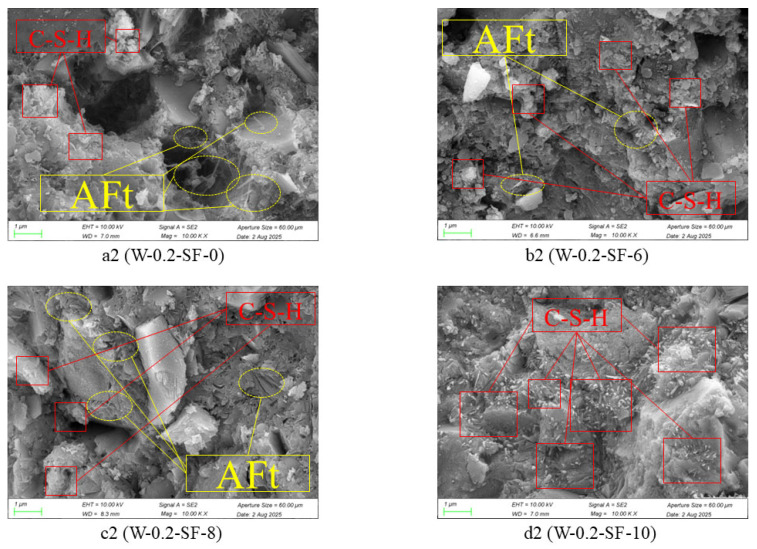
Microstructure at 7 Days.

Figure 8 further illustrates these microstructural differences: the high-SF mixture (W-0.2-SF-10) displayed substantially more C-S-H gel formation with enhanced fibrous/network-like structures that were more uniformly distributed, consequently creating a denser overall matrix. This phenomenon can be attributed to SF’s high reactivity enabling rapid early-age participation in C-S-H formation, coupled with its particle-packing effect that enhances microstructural density. These microstructural observations provide a fundamental explanation for the compressive strength trend observed in mechanical testing—the increasingly dense and continuous microstructure with higher SF content establishes a solid foundation for strength development.

The primary crystalline product of cement hydration is calcium hydroxide (CH). Silica fume (SF) participates in a secondary hydration process (pozzolanic reaction) to form C-S-H gel, as described by the reaction: CH + SiO_2_ + H_2_O → C-S-H. The hydration rate of GGBS is significantly lower than that of SF during early ages. Consequently, variations in C-S-H gel content directly reflect the differences in hydration activity between SF and GGBS.

**Figure 9:**
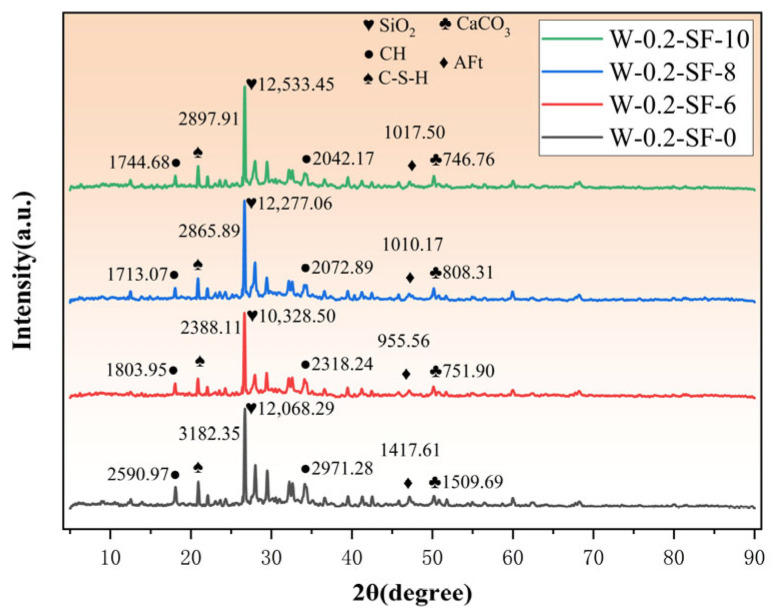
X-ray Diffraction (XRD) Patterns at 3 Days.

The 3-day XRD test results are shown in Figure 9. Analysis of the 3-day XRD patterns reveals that as the SF content increases (6%, 8%, 10%) and the GGBS content decreases (24%, 22%, 20%), the intensity of the characteristic C-S-H diffraction peak (d = 0.43 nm) progressively increases. This indicates greater C-S-H gel formation in mixtures with higher SF and lower GGBS content, confirming SF’s higher early-age hydration reactivity. SF rapidly consumes CH through the pozzolanic reaction to generate additional C-S-H, while GGBS contributes minimally to early-age C-S-H formation due to its slower reaction kinetics.

**Figure 10:**
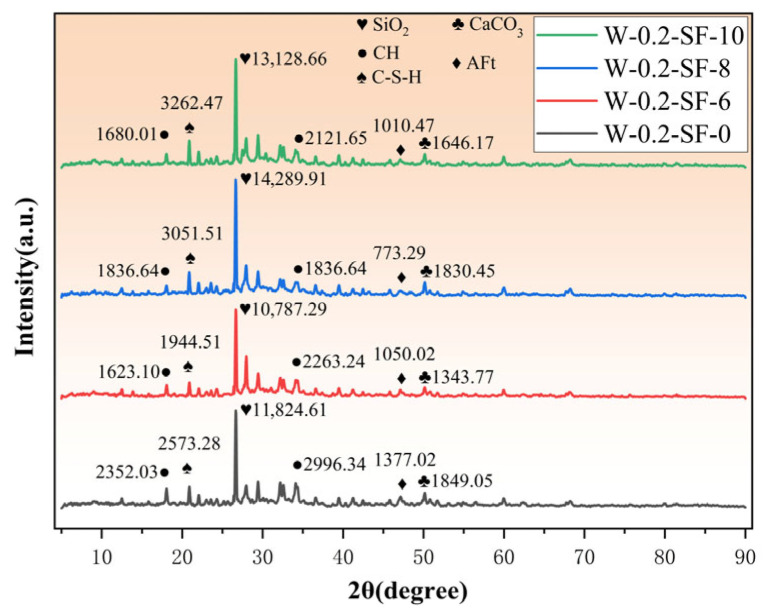
X-ray Diffraction (XRD) Patterns at 7 Days.

The 7-day XRD test results are shown in Figure 10. The 7-day XRD patterns show increased intensity of the characteristic C-S-H peaks compared to 3 days for all mixtures, indicating ongoing hydration and continuous C-S-H formation. The trend remains consistent with the 3-day results: C-S-H peak intensity escalates with increasing SF and decreasing GGBS content. This persistence occurs because SF’s hydration continues actively, and although GGBS hydration degree improves by 7 days, its overall reaction rate remains lower than SF’s. Thus, mixtures with higher SF content maintain their advantage in C-S-H production.

These results validate the proposed mechanism that faster moisture consumption drives autogenous shrinkage with increasing SF and decreasing GGBS content: SF’s higher reactivity generates more C-S-H gel, accelerating water consumption, while GGBS’s delayed reaction contributes less to early moisture depletion. This explanation aligns perfectly with the observed phenomenon of accelerated moisture consumption with higher SF content in the autogenous shrinkage tests. This monotonic increase, while failing to reveal a singular “optimum” point for C–S–H formation, represents precisely the key mechanistic finding of this study. It quantitatively corroborates the dominant role of silica fume in promoting pozzolanic reaction and C–S–H generation at the microstructural level. Hence, the value of this research lies not in identifying a peak value, but in providing the necessary quantitative relationships to support a holistic, performance-based optimization that integrates both microstructural development and its macro-scale consequences.

## 4. Conclusions

Based on comprehensive testing including compressive strength, drying shrinkage, autogenous shrinkage, and hydration heat analysis, this study investigating the mechanism by which GGBS-silica fume blends inhibit early-age autogenous shrinkage cracking risk in low water-to-binder ratio cement-based materials reaches the following conclusions.

(1) Sequential Strength Contribution of SCMs: The development of compressive strength is governed by the distinct reaction timelines of the supplementary cementitious materials. The data confirm that SF primarily enhances early-age strength through accelerated hydration and microstructure densification, as evidenced by the significantly faster strength growth rate in mixes with 6% SF within the first 7 days. Conversely, the comparable long-term strength across blends with higher GGBS content demonstrates its pivotal role in providing sustained strength development via secondary hydration, indicating a complementary, time-dependent contribution mechanism.

(2) Phase-Specific Synergy in Shrinkage Mitigation: The interaction between GGBS and SF on drying shrinkage is not constant but phase specific. The key finding is the identification of a synergistic inhibition phase during 7–12 days, where the binary blend resulted in a lower shrinkage rate than theoretically expected from individual effects. This reveals that the synergy is most effective during a critical window corresponding to the transition between early SF-dominated and later GGBS-influenced hydration, providing a targeted timeframe for shrinkage control.

(3) A Mechanistic Framework for Autogenous Shrinkage Control: Autogenous shrinkage is governed by a coupled mechanism where SF and GGBS play opposing roles. The results establish that SF acts as a “shrinkage-promoting component,” directly linking its proportion to increased early-age (≤7 days) autogenous shrinkage. In contrast, GGBS functions as a “shrinkage-mitigating component,” as its delayed hydration effectively buffers the rapid shrinkage induced by SF. This clarifies that the net early-age shrinkage is not a simple sum but a result of the dynamic competition between these two processes, offering a clear principle for mix design: balancing SF content against sufficient GGBS to counteract its shrinkage effect

(4) Synergistic Regulation of Hydration Kinetics Defines Processability Window: The GGBS-SF combination synergistically regulates hydration kinetics, primarily by extending the induction period. A critical inference from the data is that different dosage pairs (e.g., 8%/22% and 10%/20% of SF/GGBS) can yield an identical induction period. This demonstrates a quantifiable balance where the accelerating effect of SF and the retarding effect of GGBS can be proportionally tuned to achieve a desired hydration timeline. Therefore, controlling this ratio is essential for designing a practical time window for concrete placement and early-stage stress mitigation before the onset of rapid strength gain.

## 5. Limitations

Although this study has completed experiments on autogenous shrinkage, heat of hydration, compressive strength, etc., using binary blends of silica fume (replacing cement at 0%, 6%, 8%, and 10%) and slag (replacing cement at 0%, 24%, 22%, and 20%) under a fixed water-to-binder ratio of 0.2, further research involving a wider range of water-to-binder ratios is still necessary. This is because concrete shrinkage deformation is more significant under low water-to-binder ratios, and the water-to-binder ratio itself has a substantial influence on shrinkage. Therefore, experiments with multiple water-to-binder ratios are required. Additionally, since different replacement rates of silica fume and slag significantly affect concrete shrinkage, subsequent studies should also include experiments with varying replacement rates.

## Figures and Tables

**Table 1:** Chemical Composition of Cement.

Chemical Composition	CaO	SiO_2_	Al_2_O_3_	Fe_2_O_3_	SO_3_	Na_2_O	MgO	(LOI)/%
%	63.92	21.34	4.86	3.56	2.87	0.56	1.82	1.69

**Table 2:** Basic Physical Properties of Cement.

Specific Surface Area (m^2^/Kg)	Standard Consistency/(% by Mass of Cement)	Initial Setting Time/min	Final Setting Time/min	Soundness	Flexural Strength/MPa		Compressive Strength/MPa	
					3 d	28 d	3 d	28 d
384	27.1	172	254	Pass	5.5	8.7	27.1	46.9

**Table 3:** Chemical Composition of GGBS (%).

GGBS	CaO	SiO_2_	Al_2_O_3_	SO_3_	MgO	Fe_2_O_3_	TiO_2_	K_2_O	Na_2_O
%	39.30	34.53	12.73	0.40	8.27	1.93	0.90	0.80	0.30

**Table 4:** Chemical Composition of SF (%).

SF	CaO	SiO_2_	Al_2_O_3_	SO_3_	MgO	Fe_2_O_3_	TiO_2_	K_2_O	Na_2_O
%	0.29	87.50	2.15	0.52	3.86	0.70	1.11	3.31	0.56

**Table 5:** Properties of Superplasticizer.

PH	Water Reduction Rate/%	28-Day Shrinkage Ratio/%	Air Content/%	Difference in Final Setting Time (t)		Compressive Strength Ratio			
				Initial Setting	Final Setting	1 d	3 d	7 d	28 d
6.8	47	102	2.0	+25	+20	186	179	175	168

**Table 6:** Mixture Proportions.

Specimen Number	Cementitious Material			Aggregate	
	Cement (%)	SF (%)	GGBS (%)	Fine Aggregate (kg/m^3^)	Coarse Aggregate (kg/m^3^)
W-0.2-SF-0	100	0	0	860	1050
W-0.2-SF-6	70	6	24		
W-0.2-SF-8		8	22		
W-0.2-SF-10		10	20		

## Data Availability

The original contributions presented in this study are included in the article. Further inquiries can be directed to the corresponding author.
